# Multicomponent Lifestyle Interventions During Colorectal Cancer Surveillance: A Systematic Review

**DOI:** 10.3390/cancers18121906

**Published:** 2026-06-11

**Authors:** Meseret Derbew Molla, Erin L. Symonds, Jean M. Winter, Norma B. Bulamu, Melkalem Mamuye Azanaw, Molla M. Wassie

**Affiliations:** 1 Flinders Health and Medical Research Institute, College of Medicine and Public Health, Flinders University, Bedford Park, SA 5042, Australia; 2 Department of Biochemistry, School of Medicine, College of Medicine and Health Sciences, University of Gondar, Gondar P.O. Box 196, Ethiopia; 3 Gastroenterology Department, Flinders Medical Centre, Southern Adelaide Local Health Network, Bedford Park, SA 5042, Australia; 4 School of Public Health, College of Medicine and Health Sciences, Debre Tabor University, Debre Tabor P.O. Box 272, Ethiopia

**Keywords:** lifestyle, behavioural change, quality of life, randomised control trial, colorectal neoplasia

## Abstract

**Background**: Modifiable lifestyle factors may contribute additively to colorectal cancer (CRC) risk in individuals who already have non-modifiable risk factors, such as prior colorectal neoplasia or significant family history of CRC. However, the impact of multicomponent lifestyle interventions (such as dietary modification, physical activity, and counselling) on behavioural modification, risk of colorectal neoplasia, and quality of life (QoL) in this population has not yet been systematically reviewed. **Aims**: The primary aim was behavioural change (change in body weight, diet, physical activity, sedentary lifestyle, smoking, and alcohol consumption). The secondary aim was colorectal neoplasia outcomes, including the incidence of precancerous lesions and/or cancer and CRC mortality/survival, and QoL, including specific domains. **Methods**: This review was conducted following the Cochrane guidelines for Systematic Reviews of Interventions. Both randomised and non-randomised studies assessing the effect of multicomponent lifestyle interventions on behavioural modification, risk of colorectal neoplasia, mortality, and quality of life in people at above-average risk of CRC were included. Medline/Ovid, Cochrane Library, Web of Science, and Scopus were searched. Screening, data extraction, and risk of bias assessment were independently performed by two reviewers using the revised Cochrane Risk of Bias (RoB) tools. **Results**: Of the 4174 studies screened, 10 interventional studies were eligible for inclusion, which had outcomes for behavioural change or quality of life. No interventions assessed neoplasia risk or mortality outcomes. Multicomponent lifestyle interventions mainly targeting diet and physical activity, delivered via a telephone-based or health coaching approach, showed positive effects on healthy behaviours and quality of life compared with usual care, although some studies reported inconsistent results. **Conclusions**: There is emerging evidence that multicomponent lifestyle interventions may offer beneficial effects on practicing healthy behaviours and improving QoL for individuals at above-average risk for CRC and undergoing colonoscopy surveillance.

## Simple Summary

Bowel cancer is the third most commonly diagnosed cancer and the second leading cause of cancer-related death worldwide. It is a lifestyle-related cancer and can be prevented by lifestyle modifications. However, people who have a prior history of colorectal neoplasia (both bowel cancer and precancerous polyps) and a family history of bowel cancer are at higher risk of bowel cancer and are, therefore, grouped as being at above-average risk for bowel cancer. For this population, those who are already at above-average risk for bowel cancer, interventions involving more than one lifestyle factor can help to reduce colorectal neoplasia outcomes (incidence or mortality) and bring behavioural changes to improve their health and quality of life. Therefore, this study aimed to assess the effect of multicomponent lifestyle interventions on people who are at increased risk of bowel cancer. The findings of the study will help to target multiple lifestyle interventions rather than a single-component lifestyle intervention.

## 1. Introduction

Colorectal cancer (CRC) is one of the leading causes of morbidity and cancer-related mortality worldwide [1]. CRC is becoming a public health concern globally, with the incidence and mortality rate increasing in low- and middle-income countries [2]. CRC often develops from precancerous polyps, such as conventional adenomas or serrated lesions [3,4]. Different strategies, including CRC screening [5] and colonoscopy-based surveillance, have been implemented to reduce the burden of CRC [6]. In many developed countries, CRC screening programmes are implemented for individuals at average risk (asymptomatic adults with age > 45 years) using either faecal immunochemical test or colonoscopy, which have shown a proven impact in reducing the incidence and mortality of CRC [7]. However, the average screening rate remains low, and it is below 50% in many countries [8]. The Centres for Disease Control and Prevention (CDC) established the CRC Control Programme (CRCCP) to increase screening rates among adults aged 50–75 years, particularly in areas with low screening rates, by partnering with primary care clinics and supporting the implementation of combined strategies or evidence-based interventions (EBIs) [9]. Key EBIs include client or provider reminders, provider assessment and feedback, and efforts to reduce structural barriers to screening, supplemented with additional strategies such as patient navigation, professional development, and small media [9]. It showed that these approaches, particularly the combination of multiple EBIs (≥3 components) along with additional strategies, had the greatest increases in clinic-level screening rates [10]. Client reminders (8.0%) among the EBIs and CRC screening (8.4%) among all components were the interventions most associated with an increased screening rate [10]. For individuals at above-average CRC risk, including individuals with a significant family history of CRC or a personal history of colorectal neoplastic polyps or cancer, surveillance colonoscopy is recommended for early detection of CRC or recurrence, and removal of any precancerous polyps [6].

The progression of precancerous polyps to CRC is a slow process, usually taking more than 10 years [11]. However, the risk of developing CRC is largely dependent on both non-modifiable hereditary and environmental lifestyle factors [12]. Around 40% of CRC risk is linked to modifiable lifestyle factors, including poor dietary habits, lack of physical activity, tobacco use, excessive alcohol intake, and high body mass index [13]. Although the incidence of CRC has declined or has plateaued in most developed countries, the sharp rise in CRC cases in low- and middle-income countries is likely due to a global trend toward unhealthy lifestyles [2]. According to Global Burden of Disease estimates 2023, the incidence of CRC increased substantially between 1990 and 2023 in low- and middle-income countries, particularly in countries in Latin America, Southeast Asia, the Caribbean, North Africa, and sub-Saharan Africa [14]. Although the exact drivers of the rising incidence of CRC in low- and middle-income countries are not fully understood, unhealthy lifestyle behaviours are likely to play a significant contributory role [15]. Modifiable risk factors interact with non-modifiable factors to further increase CRC risk [16]. In addition to unhealthy lifestyles being associated with precancerous polyp and CRC development, they are also linked with poorer quality of life (QoL) [17] and increased mortality [18]. CRC survivors face an increased risk of mortality associated with unhealthy behaviours such as poor dietary habits [19], smoking [20], physical inactivity [21], and obesity [22]. These individuals also have significant psychological and physical challenges that can severely impact their overall QoL [23,24], and poor lifestyle behaviours may further compound this. It is therefore important to address the effect of multicomponent lifestyle interventions on behavioural change, CRC outcomes, and QoL in people at increased risk of CRC. Multicomponent lifestyle interventions refer to the intervention of at least two of the following lifestyle factors: smoking, physical activity, diet, alcohol consumption, and weight maintenance [18]. In contrast, a single-component lifestyle intervention involves targeting only one of the lifestyle factors [17].

Previous systematic and meta-analyses of observational studies suggest that a one-component lifestyle change, such as regular physical activity, maintaining a healthy weight, eating a healthy diet, and avoiding tobacco and alcohol, can help to reduce the burden of CRC, both the risk and mortality [25,26,27], and improve QoL [17]. Recent systematic reviews and meta-analyses have also assessed the effect of a multicomponent lifestyle intervention (intervention of more than one lifestyle factor) on CRC outcomes. For instance, Yu et al., 2022, in their review of 28 observational studies, found that adherence to multicomponent healthy lifestyle behaviours is associated with a reduced risk of colorectal adenoma, CRC incidence, and CRC-specific mortality among healthy populations or people after CRC treatment [18]. Similarly, another meta-analysis among the general population and CRC survivors focusing on cohort studies reported that individuals who follow multiple healthy lifestyle practices have a lower risk of overall cancer incidence and mortality [28]. A recent systematic review of five studies involving 719 participants also explored the impact of multicomponent lifestyle interventions on QoL among CRC survivors [29]. However, the findings regarding QoL improvements were inconsistent and did not specifically assess populations with a family history of CRC or those affected by precancerous polyps. A systematic review and meta-analysis of 15 studies also demonstrated that the implementation of behavioural change interventions improved diet, increased physical activity, and reduced sedentary behaviours [30]. However, the review was limited to CRC survivors and did not specifically evaluate interventions involving multiple lifestyle factors. As lifestyle factors do have a synergetic effect, assessing the effect of multicomponent lifestyle interventions on behavioural change, colorectal neoplasia outcomes, and QoL in people at above-average risk offers greater value than assessing single lifestyle interventions in isolation.

The prior reviews on the effect of multicomponent lifestyle intervention on behavioural change, CRC outcomes, and QoL have mainly included observational studies. One systematic review assessed the effect of multicomponent lifestyle intervention on the improvement of QoL, but limited the cohort to CRC survivors and did not include studies with individuals with a significant family history of CRC or personal history of precancerous polyps [29]. Although another systematic review has summarised the impact and feasibility of lifestyle interventions on behavioural change, colorectal neoplasia outcomes, and QoL in patients diagnosed with CRC [31], there remains a gap in the literature regarding multicomponent lifestyle intervention effects in individuals at above-average risk of CRC, including for CRC survivors. There is a growing number of primary studies that have assessed the effect of multicomponent lifestyle interventions on behavioural change, colorectal neoplasia outcomes, and QoL in individuals at above-average risk of CRC. The present study therefore aimed to summarise the evidence on how multicomponent lifestyle interventions influence behavioural change, the risk of colorectal outcomes (incidence and/or mortality), and QoL in above-average CRC risk populations.

## 2. Methods

This systematic review protocol was registered with Prospero (CRD420251046134) and also published in a peer-reviewed journal [32]. It was conducted according to the Cochrane guidelines for Systematic Reviews of Interventions [33] and adhered to the Preferred Reporting Items for Systematic Reviews and Meta-Analyses (PRISMA) [34]. The review followed the Population, Intervention, Comparator and Outcome (PICO) Framework.

### 2.1. Eligibility Criteria

Any clinical trial studies, including randomised controlled trials (RCTs), non-randomised trials such as quasi-experimental studies and interrupted time series, and pragmatic trials, focusing on interventions in real-world clinical settings rather than controlled environments, were included. Clinical trials from all settings, such as hospitals, communities, and long-term care facilities, were included. Pilot clinical trial studies were also considered. The study population included adults (aged 18 years or older) who were at above-average CRC risk due to a significant family history of CRC and/or a personal history of colorectal neoplasia (precancerous lesions and/or CRC). The intervention included at least two concurrent lifestyle interventions, such as diet, weight maintenance, physical activity or exercise, smoking cessation, or alcohol reduction. Comparator (control) groups either had no lifestyle intervention, a single lifestyle intervention, or usual care. The primary outcome of interest was behavioural change (change in body weight, diet, physical activity, sedentary lifestyle, smoking, and alcohol consumption). The secondary outcomes were colorectal neoplasia (precancerous lesions and/or cancer) incidence, CRC mortality/survival, and QoL, including health-related quality of life. Studies that examined the effect of a single lifestyle factor as the intervention were excluded. Grey literature, reviews, protocols, conference abstracts, non-interventional studies, and studies not published in English were excluded.

### 2.2. Search Strategy and Selection

The following four databases were searched to find relevant studies from inception to 11 April 2025: Medline/Ovid, Cochrane Library, Web of Science, and Scopus. MeSH terms and keywords related to the study objectives were used both independently and in combination through Boolean operators to search for articles. A systematic master search was developed using the Medline/Ovid platform (Supplementary Table S1) and interpreted into other databases as appropriate.

Identified studies were imported into EndNote for management and then into Covidence for screening. Duplicates were removed automatically using Covidence or manually for those undetected. Studies were screened (title, abstract, and full text) by two independent reviewers (MDM and MMA). Any discrepancies were resolved through consensus or discussion with all authors.

### 2.3. Data Extraction

Two independent reviewers (MDM and MMA) extracted relevant information from the included studies. Data was extracted using Microsoft Excel, using a modified Cochrane data-extraction tool. Characteristics of the study, such as first author, year of publication, country, setting, characteristics of study participants (number of participants, age, sex, selection criteria, withdrawal/losses to follow-up, and subgroups), outcomes (point estimates and measures of variability and frequency counts for dichotomous variables), and any relevant pieces of information were extracted. Disagreements were resolved by consensus or discussion with the senior authors (ELS, JMW, NB, and MMW).

### 2.4. Risk of Bias

Two independent reviewers (MDM and MMA) assessed the methodological quality of the included studies using the revised Cochrane Risk of Bias (RoB2) Tools for randomised trials [35] and Risk Of Bias In Non-randomised Studies—of Interventions (ROBINS-I) [36]. Any disagreement was resolved by consensus or discussion with the senior authors (ELS, JMW, NB, and MMW). The revised RoB2 tool covers five domains: (1) bias arising from the randomisation process, (2) bias due to deviations from intended interventions, (3) bias due to missing outcome data, (4) bias in the measurement of outcomes, and (5) bias in the selection of the reported results [35]. Each domain has sub-item measurements, and the total risk of bias for each domain, as well as the overall score, was categorised into one of three groups: low risk of bias, some concerns, or high risk of bias [35].

### 2.5. Data Synthesis

The results were systematically synthesised and presented both quantitatively and qualitatively. A structured thematic narrative review that provided an in-depth interpretation of the data, highlighting consistent patterns, contextual factors, and variations in study design, population characteristics, and intervention components was completed. Outcomes were mapped into behavioural change (including change in physical activity, diet, body weight, cigarette smoking, alcohol consumption, and sedentary lifestyle), risk or mortality of colorectal neoplasia, and QoL.

### 2.6. Amendment from the Published Protocol

The initial plan was to conduct a meta-analysis; however, this was not feasible since the included studies were heterogeneous regarding the types of lifestyle interventions, measurement of effect, and the follow-up period. The heterogeneity of the included studies includes differences in the duration of intervention (varies from 6 weeks to 12 months), intervention delivery modes (varies from simple counselling to coaching), variation in control groups (varies from a standardised guideline approach or usual care to intervention of one lifestyle factors), outcome measurement tools (measurement of each outcome was different), and baseline participant characteristics (only one study focused on family history of CRC, whereas others focused on CRC survivors). Subsequently, subgroup analysis to identify the dose–response effect of the multicomponent lifestyle interventions on behavioural change, colorectal neoplasia risk, mortality, and quality of life was not performed. A subgroup analysis by location or site of tumour (colon vs. rectal) and sex (male vs. female) was not performed due to a lack of relevant information provided in the included studies.

### 2.7. Patient and Public Involvement

Patient or public involvement was not applicable for this study in the design, conduct, reporting, or dissemination plans.

### 2.8. Ethics Approval

Ethical approval was not needed as this systematic review is entirely focused on existing literature.

## 3. Results

### 3.1. Descriptive Characteristics of the Study

**Figure 1:**
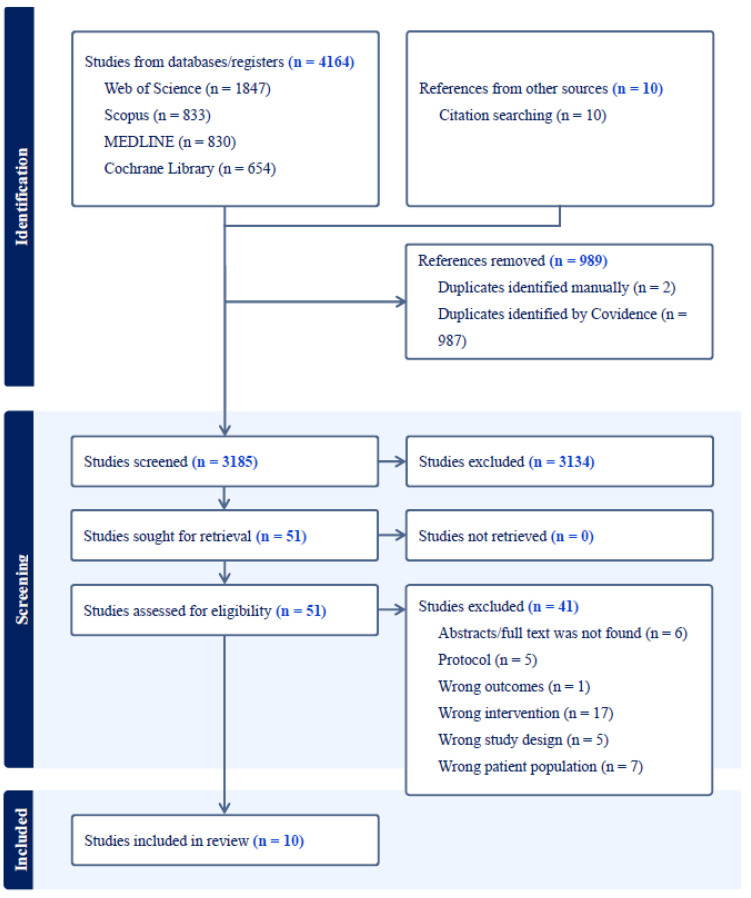
PRISMA flowchart of article screening.

About 4164 articles from four databases and 10 manually searched articles, which gives us a total of 4174 articles, were imported into Covidence for article screening (Figure 1). Of these, 989 duplicates were removed, and 3185 titles and abstracts were screened. Of these, 51 full-text articles were assessed for eligibility, with 10 articles included in this review. Studies that underwent full-text review but were not included in the final analysis and the reasons for their exclusion are listed in Supplementary Table S2.

Five (50%) of the included studies were conducted in Australia [37,38,39,40,41], two in China [42,43], and two in the United Kingdom [44,45], while one was conducted in Taiwan [46]. The majority (9/10) of studies were RCTs (two were pilot studies) [40,44], and one study was a non-randomised feasibility trial [45]. Nine studies were conducted among CRC or colon cancer survivors, while only one was conducted among first-degree relatives of CRC survivors [40]. For all RCTs, the intervention and control groups were allocated using a 1:1 ratio (Table 1). All studies, except the study conducted in Taiwan [46], reported being supported by sponsors/funders (Supplementary Table S3).

### 3.2. Types of Multicomponent Interventions and Study Outcomes

Of the 10 studies that applied multicomponent lifestyle interventions, three assessed QoL outcomes [37,39,42], three assessed health behaviour change [40,41,43], and four assessed both QoL and behaviour change [38,44,45,46] (Table 2).

In four studies [37,38,39,41], the intervention was a telephone-delivered health behaviour change programme that included multicomponent lifestyle changes compared with usual care. The multicomponent health behaviours addressed in telephone sessions included a specific acceptance and commitment to therapy processes related to lifestyle behaviours (values, mindfulness, diffusion, acceptance, and committed action), as well as strategies to enhance improvement in health behaviours consistent with guideline recommendations. One study included health coaching that provided educational content, including practical behavioural change strategies, by focusing on moderate-to-vigorous physical activity, reduction in television viewing, diet, alcohol intake, body mass index (BMI), and waist circumference, compared with standard care [40]. Three studies focused on dietary and physical activity interventions [42,43,44], while the only non-randomised study implemented an intervention that included education on smoking cessation, increasing moderate physical activity, and brief interventions on alcohol and weight management [45] (Table 2).

Seven studies assessed the impact of multicomponent lifestyle interventions on QoL [37,38,39,40,42,44,46]. Five studies evaluated changes in body weight, measured using BMI [38,40,41,44,45], one used the waist-to-hip ratio [44], and another used waist circumference [40]. Of these studies, one non-randomised trial reported using change in median BMI [45] while others used a mean difference in BMI. Five studies also examined how multicomponent lifestyle interventions influenced changes in physical activity [38,40,41,43,44], while one study focused on changes in sedentary behaviour [41]. Four studies investigated the impact of multicomponent interventions on dietary changes [38,40,43,44]. None of the studies investigated the impact of multicomponent lifestyle interventions on the risk of colorectal neoplasia and associated mortality among the above-average CRC risk population (Table 2).

### 3.3. Risk of Bias Assessment of the Studies

**Figure 2:**
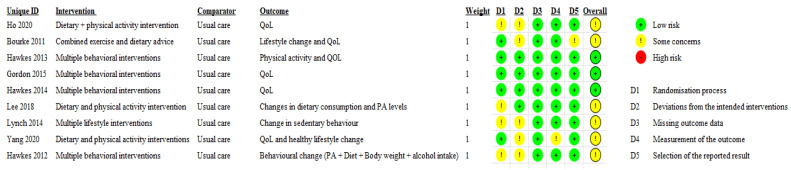
ROB2 quality assessment of the included studies. Unique ID is in the format of [Author Year]. QoL—quality of life; PA—physical activity [37,38,39,40,41,42,43,44,46].

Of the total included studies, three were graded as low risk of bias [37,38,39], while the remaining studies had some concerns in the overall risk of bias assessment. Five studies in domain two, which assessed deviations from the intended interventions, and four studies in domain one, which evaluated the randomisation process, raised some concerns. All studies were graded as low risk in domain three, which assessed the absence of outcomes, while only one study in domains four and five was graded as having some concerns. None of the studies were graded as high risk in each domain or overall assessment (Figure 2).

### 3.4. Effect of Multicomponent Lifestyle Interventions on Incidence, Recurrence, or Mortality of Colorectal Neoplasia

As displayed in Table 3, none of the eligible studies for this review reported on the effect of multicomponent lifestyle interventions on incidence, recurrence, or mortality of colorectal neoplasia.

### 3.5. Effect of Multicomponent Lifestyle Interventions on Behavioural Change

#### 3.5.1. Change in Body Weight

A total of 5/10 studies assessed change in body weight with a multicomponent lifestyle intervention, with inconsistent findings. As presented in Table 3, one study reported that multicomponent lifestyle interventions helped to reduce body weight, which was measured using the waist-to-hip ratio (mean difference (MD) = −0.05, 95% CI −0.1 to −0.01), but not BMI (MD = −0.4, 95% CI −0.8 to 0.1) [44]. However, Hawkes et al., 2012, had found that multicomponent lifestyle intervention reduces both BMI (MD = −1.4, 95% CI −2.3 to −0.5) and waist circumference (MD = −5.1, 95% CI −8.3 to −2) [40]. On the other hand, Lynch et al., 2014, reported that multicomponent lifestyle intervention improves BMI after 6 months of follow-up (MD = −0.69, 95% CI −1.28 to −0.1) and after 12 months of follow-up (MD = −1.4, 95% CI −2.0 to −0.8) for those who had BMI < 30 kg/m^2^ at baseline, but it had no effect for those who had BMI ≥ 30 kg/m^2^ [41]. One other study also found that a decrease in body weight was observed due to multicomponent lifestyle intervention at 6 months (0.5 kg/m^2^; *p* = 0.035) and 12 months (0.9 kg/m^2^; *p* = 0.001) [38].

#### 3.5.2. Change in Dietary Intake

Following multicomponent lifestyle intervention and subsequent follow-up, improvements in healthy dietary intake remain inconclusive. While one study reported a significant reduction in dietary starch consumption in both the intervention and control groups [44], other studies observed additional improvements in the intake of vegetables, fruits, and reductions in red and processed meat consumption (Table 4). Lee et al., 2018, found that after a multicomponent lifestyle intervention and follow-up for 6, 12, 18, and 24 months, there were consistent reductions in intake of red and processed meat and refined grain intake [43]. On the other hand, a pilot study among individuals with a significant family history of CRC reported that after 6 weeks of follow-up, there was a one reduction in processed meat intake (MD = −1.2, 95% CI −1.8 to −0.5) and increase in vegetables intake (MD = 1.0, 95% CI 0.4 to 1.6), but no changes in the intake of red meat (MD = 0.02, 95% CI −0.6 to 0.6) or fruits (MD = of 0.3, 95% CI −0.3 to 0.9) [40]. Additionally, Hawkes et al., 2013, reported using the adjusted between-group difference in mean change and observed a reduction on total fat consumption at 6 months (8.5%; *p* = 0.001) and 12 months (7.0%; *p* = 0.006), saturated fat at 6 months (3.5%; *p* = 0.002) and 12 months (2.8%; *p* = 0.016), and vegetables at 6 months (0.4 servings per day; *p* = 0.001), but not at 12 months (2%; *p* = 0.139); however, they did not observe any significant differences in fruit, fibre, or alcohol intake at 6 or 12 months of follow-up [38].

#### 3.5.3. Change in Physical Activity and Sedentary Behaviours

Participants who engaged in multicomponent lifestyle interventions spent significantly more time in moderate-to-vigorous physical activities, improved exercise tolerance, and reduced sedentary behaviours, such as prolonged sitting or television viewing, compared to those receiving standard care (Table 5). One study observed a significant positive effect of a multicomponent lifestyle intervention on exercise tolerance (*p* = 0.01) but not on maximum voluntary torque (*p* = 0.127) [44]. Another study also observed a significant improvement in moderate but not in vigorous physical activity at 12 months (*p* = 0.023) following a multicomponent lifestyle intervention. Additionally, both moderate and vigorous physical activity were improved at 6 months following a multicomponent lifestyle intervention [38]. A pilot study among individuals with a significant family history of CRC also observed a significant effect on the mean change in moderate-to-vigorous physical activity at 12 weeks following a multicomponent lifestyle intervention (*p* < 0.001) [40]. One study observed that a multicomponent lifestyle intervention significantly reduced sedentary behaviours such as time spent watching television/videos (h/d), screen time (h/d), and total sedentary time (h/d) [41].

#### 3.5.4. Effect of Multicomponent Lifestyle Intervention on the Quality of Life

Across the included studies, the impact of multicomponent lifestyle interventions on generic QoL or health-related quality of life (HRQoL) showed generally positive trends. Four included studies reported the generic QoL or HRQoL using FACT-C, SF-6D, and SF-36 scores [37,39,42,44]. None of these included studies showed a statistical improvement of overall QoL after multicomponent lifestyle interventions. However, one of the included studies reported a significant improvement of QoL measured using FACT-G, which is a fatigue-specific QoL improvement, suggesting domain-specific rather than global benefits for the included studies [44] (Table 6).

##### Physical Domain

Six studies assessed the effect of multicomponent lifestyle interventions on the physical domain of QoL using SF-12 PCS, SF-36 PCs, FACT physical well-being, and WHOQOL physical health [37,38,39,40,42,46]. All included studies showed a consistent improvement in the physical domain of QoL in both groups, with slightly better improvements in the intervention domains. However, only one study observed a significant improvement in physical functioning, bodily pain, general health, vitality, and physical HRQoL in the intervention group compared to the control (*p* < 0.05) [40] (Table 6).

##### Psychological/Emotional Well-Being Domain

Six studies were included in this domain, measured using SF-12 MCS, HADS (anxiety/depression), and emotional well-being (FACT, WHOQOL) [37,38,39,40,42,46]. None of them showed a statistically significant effect of multicomponent lifestyle intervention on the psychological/emotional well-being or mental health domain of QoL. A study on a population with a significant family history of CRC reported an improvement of the mental health domain of HRQoL, but it was not a statistically significant effect (*p* = 0.16) [40] (Table 6).

##### Social Domain

Three studies assessed the effect of multicomponent lifestyle interventions on the social domain of QoL, which was measured using SF-36, FACT social well-being, and WHOQOL social relationships [39,40,46]. All reported a statistically insignificant improvement of social well-being (Table 6).

## 4. Discussion

This review systematically evaluated the available published literature investigating the effect of multicomponent lifestyle interventions on behavioural modifications, in addition to the risk of colorectal neoplasia or mortality and QoL. We found that multicomponent lifestyle interventions had a positive relationship with reductions in body weight, increments in physical activity, reductions in sedentary behaviour, improvements in healthy diet intake, and QoL. However, the findings were not consistent across the individual outcomes. Additionally, we found there were no published studies that investigated the impact of multicomponent lifestyle intervention on the incidence of CRC, adenoma, or associated mortality.

Studies applying multicomponent lifestyle interventions showed a positive effect on body weight changes, but the effect was not consistent across different weight measurement methods and baseline weights [38,40,41,44,45]. For example, BMI is a gross measurement of body size and may not be easily modified within a short intervention period, while the waist-to-hip ratio and waist circumference are more sensitive measurements of adiposity [47]. The effect of multicomponent lifestyle interventions on change in body weight relative to the starting body weight could also be partially explained by adherence to interventions for those who are already overweight, as adherence often declines after the initial intervention phase, leading to weight regain [48]. Additionally, these individuals may not lose weight after multicomponent lifestyle interventions for several interconnected reasons, specifically due to metabolic adaptation and hormonal factors [48]. Metabolic adaptation may make weight loss harder and can even lead to weight regain despite continued adherence to the programme; for example, if the intervention involves lowering energy intake, the body may respond by lowering its basal metabolic rate to conserve energy rather than weight loss [48]. Hormonal imbalances, such as leptin and insulin resistance, may also counteract weight loss efforts by affecting the hunger, satiety, and energy expenditure physiology, making it difficult to sustain calorie loss [48]. However, none of the included studies in this review point out that metabolic adaptation and hormonal factors are possible mechanisms to limit weight loss after multicomponent lifestyle intervention for those who are already overweight; thus, further studies are warranted to confirm this. Although there are no similar systematic reviews on the impact of multicomponent lifestyle interventions on the reduction in body weight, a systematic review and meta-analysis of 12 randomised studies that investigated all types of behaviour change interventions (either single or in combination) did not find a significant effect of behavioural change techniques on body weight and BMI in CRC survivor patients [30].

This review found inconclusive results on the effect of multicomponent lifestyle interventions on healthy dietary intake. The main goal of dietary interventions to prevent CRC is to increase consumption of fibre, whole grains, fruit, vegetables, and seafood while reducing the consumption of red and processed meat, refined grains, and alcohol [49]. In this review, we found that multicomponent lifestyle interventions (multiple behaviour-change interventions or dietary and physical activity interventions) improved intake of refined grains, vegetables, and fruits and reduced intake of red and processed meat [38,43]. On the other hand, a pilot study among individuals with a significant family history of CRC reported no improvement in red meat or fruit intake after a 6-week follow-up of multicomponent lifestyle interventions (healthy lifestyle education) [40]. This difference might be due to the study population’s risk level difference for CRC and how they adhere differently. For example, having a family history of CRC is different from a personal experience of having CRC, and therefore adherence may be stricter in the cohorts that were found to have a significant effect. The discrepancy of the findings may also be due to the differences in the duration of the intervention (a 6-week follow-up may be too short for behavioural changes regarding dietary intake, and may not show a statistically significant change), type of component, and number of the lifestyle interventions [40]. Our current findings are in line with the review by Wang et al., 2023, which revealed that the implementation of different behavioural change techniques had a significant impact on dietary behaviours, particularly in enhancing the frequency of fruit and vegetable consumption and improvement of dietary knowledge [30]. However, the review solely focused on CRC survivors and did not restrict the intervention group to only a multicomponent lifestyle intervention (the intervention included all types of behaviour change, including single and multicomponent), whereas our review analysed the effect of multicomponent lifestyle interventions on behavioural change among individuals at above-average risk of CRC, including CRC survivors, who had previous precancerous colorectal polyps as well as a significant family history of CRC.

The current review also found that people who engaged in multicomponent lifestyle interventions spent significantly more time in moderate-to-vigorous physical activity, improved exercise tolerance, and reduced sedentary behaviours, such as prolonged sitting or television viewing, compared to those receiving standard care. However, the findings across the included studies were inconsistent, potentially due to variations in population characteristics that influence exercise preferences and adherence, such as those who had a personal history of CRC having different exercise preferences and adherence for interventions compared to those with a family history of CRC. Therefore, it is essential to conduct comparative analyses to evaluate the effects of different demographic (e.g., age category) and clinical factors, such as baseline BMI values [50]. Although the duration of interventions varied among the included studies, improvements in physical activity resulting from multicomponent lifestyle interventions were also more evident at the six-month follow-up compared to the twelve-month post-intervention assessment. Comparable results were identified in Wang et al., 2023, which examined the impact of various behavioural change interventions, including single and multicomponent lifestyle approaches, on physical activity, and reported that patients’ activity levels at six months exceeded those observed at three or twelve months for CRC survivors [30]. The underlying cause of this variation remains unclear; however, it may be attributable to short follow-up times that fail to capture the true intervention effect, or to challenges in sustaining long-term adherence to lifestyle modifications, which could lead to a decline in physical activity improvements [31,51]. Therefore, to enhance the validity and applicability of the effect of multicomponent lifestyle interventions on physical activity and sedentary behaviour, future interventional studies should consider individual demographic and clinical profiles at baseline and adopt intervention durations.

The present review also found that multicomponent lifestyle interventions can lead to improvements in patients’ QoL and its individual components. However, these findings were not consistently observed across all studies. This inconsistency may be attributed to several factors, including variations in intervention design, duration, participant characteristics, and the QoL assessment tools used. For instance, Hawkes et al., 2014 [39], reported enhanced FACT-C scores at both 6 and 12 months after multicomponent health behaviour change interventions, and Yang et al., 2020 [46], observed an improvement in overall QoL using the WHOQOL-BREF after a 3-month follow-up period after dietary and physical activity interventions, while Ho et al., 2020 [42], noted improvements only in the SF-6D utility index after 6 months after dietary and physical activity interventions. In contrast, other studies did not find significant changes in QoL after multicomponent lifestyle intervention (multiple health behaviour change interventions or lifestyle interventions on multicomponent supervised and home-based exercise sessions and dietary advice) [38,44]. This may be partially explained by the lack of standardised measurements specific to the interventions and may obscure the true impact of multicomponent lifestyle interventions. Moreover, differences in adherence levels and the intensity of behavioural support provided could further influence outcomes. These findings underscore the need for future research to adopt harmonised methodologies and longer follow-up periods to better understand the sustained effects of lifestyle interventions on QoL in populations at above-average risk for CRC. A previous systematic review by Gielen et al., 2024 [29], of five studies that focused on the effect of multicomponent lifestyle interventions on post-treatment QoL for CRC survivors also found inconsistent results. Five of these studies are also included in the current review. However, the previous review did not specifically assess populations with a family history of CRC or those affected by precancerous polyps. While our review provides conclusive insights within the available evidence, we found no studies that examined the impact of multicomponent lifestyle interventions on QoL among individuals with a history of precancerous polyps. This highlights a notable gap in the literature and emphasises the need for targeted research in above-average-risk populations to better understand the potential benefits of lifestyle interventions for the improvement of overall QoL and its components. On the mental health domain of QoL, none of the included studies reported a statistically significant improvement due to multicomponent lifestyle interventions. This may reflect that most multicomponent lifestyle interventions included in the studies, such as physical exercise, diet, smoking, and alcohol consumption, may primarily target the physical component as compared to mental health (psychological domain). Mental health improvements are often indirect for lifestyle interventions, rather associated with social determinants such as social support, financial stability, and access to care, and mediated through other factors such as motivation, coping, or symptom severity and recovery [52,53,54]. However, these mediators were not measured in the included studies, and further studies considering the mediators and social well-being of the participants are warranted.

A key strength of this review lies in its comprehensive synthesis of evidence on the effects of multicomponent lifestyle interventions on changes in behavioural factors such as diet, physical activity, and QoL. Importantly, the review focuses on populations at above-average risk for CRC due to a history of colorectal neoplasia or family history of CRC, offering valuable insights for both clinical practice and public health strategies. Despite its contributions, the study has several limitations that warrant consideration. First, we cannot find any study that assessed the effect of multicomponent lifestyle intervention on the risk of adenoma incidence/recurrence and CRC incidence/mortality. Second, the limited scope of lifestyle intervention was utilised in the included studies, and the dose–response effect was not analysed, which may restrict the actual effect of lifestyle intervention on outcomes. Third, assessment tools for each outcome were not similar across the included studies, which made it impossible to meta-analyse and compare study findings within the included studies. Fourth, the post-intervention follow-up period was relatively short (the maximum was 24 months), which restricts the ability to assess long-term outcomes. Fifth, most included studies demonstrated a moderate risk of bias, which collectively lowers the overall strength of the evidence. Finally, this review is only focused on articles published in English and may miss potential articles published in another language, since the authors did not have resources for translation. These limitations highlight the need for more comprehensive, long-term, and methodologically rigorous studies to better understand the role of lifestyle interventions in improving behavioural, diet, and QoL change for a population at above-average CRC risk.

## 5. Conclusions

This review found that multicomponent lifestyle interventions targeting physical activity, dietary intake, smoking cessation, and alcohol consumption bring behavioural change, including an increase in physical activity levels, weight reduction, and healthy dietary choices. Additionally, we found that multicomponent lifestyle intervention improves QoL for individuals at above-average risk of CRC.

### 5.1. Clinical Practice Implications

The findings of this review suggest that it is advisable to consider the integration of multicomponent lifestyle interventions in colorectal neoplasia prevention programmes/activities to improve CRC prevention. Training of healthcare providers to give proper counselling or education on modifiable risk factors of CRC is advisable.

### 5.2. Public Health Recommendations

We recommend promoting and integrating comprehensive health education on combined healthy lifestyle behaviours, such as encouraging regular physical activity, maintaining a healthy body weight, avoiding or quitting smoking, limiting alcohol consumption, and adopting a balanced diet rich in fibre, fruits, and vegetables while reducing red and processed meat intake. Such interventions should be widely implemented at the population level, with particular emphasis on individuals at above-average risk for CRC, to limit the incidence/recurrence of colorectal neoplasia, and to improve QoL, weight maintenance, and healthy lifestyle behaviours.

### 5.3. Future Research Directions

Future studies should address the effect of multicomponent lifestyle interventions on colorectal neoplasia outcomes, including incidence of precancerous polyps, their advanced form, histopathological subtypes (adenoma vs. serrated polyps), and CRC survival/prognosis and mortality. A standardised definition of multicomponent lifestyle interventions and their optimal intervention time for individuals at above-average risk for CRC should also be covered in the future.

## Figures and Tables

**Table 1:** Characteristics of the included studies.

Study	Country	Study Design	Study Setting	Participant Characteristics	Total Sample Size	Intervention	Control
Bourke 2011 [44]	United Kingdom	Pilot RCT	Sheffield Hallam University rehabilitation facility/Northern General Hospital, Sheffield, United Kingdom	Colon cancer survivors	18	9	9
Gordon 2015 [37]	Australia	RCT	Recruited from Queensland Cancer Registry	CRC survivors	410	205	205
Hawkes 2013 [38]	Australia	RCT	Recruited from Queensland Cancer Registry	CRC survivors	410	205	205
Hawkes 2014 [39]	Australia	RCT	Recruited from Queensland Cancer Registry	CRC survivors	410	205	205
Hawkes 2012 [40]	Australia	Pilot RCT	Adults in Queensland, Australia—community setting	First-degree relatives of CRC survivors	22	22	NA
Ho 2020 [42]	China	RCT	Surgical and oncological departments of four public hospitals in Hong Kong	CRC survivors	224	Group A (Dietary + physical activity) (*n* = 55), Group B (Dietary only) (*n* = 56) and Group C (physical activity only) (*n* = 56)	Group D (Usual care control) (*n* = 56)
Lee 2018 [43]	Hong Kong, China	RCT	Surgical and oncological departments of four public hospitals in Hong Kong	CRC survivors	224	Group A (Dietary + physical activity) (*n* = 55), Group B (Dietary only) (*n* = 56) and Group C (physical activity only) (*n* = 56)	Group D (Usual care control) (*n* = 56)
Lynch 2014 [41]	Australia	RCT	Recruited from Queensland Cancer Registry	CRC survivors	410	205	205
Macleod 2018 [45]	United Kingdom	Non-randomised feasibility trial	National Health Service (NHS) Tayside	CRC survivors	22	15	NA
Yang 2020 [46]	Taiwan	RCT	National Cheng Kung University Hospital	CRC survivors	68	34	34

RCT—randomised controlled trial, CRC—colorectal cancer, QoL—quality of life, NA—not applied.

**Table 2:** Intervention and outcome characterisation of the included studies.

Study	Follow-Up Time	Duration of Intervention Details	Lifestyle Interventions	Description of Intervention	Control Groups/Comparator	Specific Measurement of Outcomes
Bourke 2011 [44]	6–24 months	12-week multicomponent lifestyle intervention, in the first 6 weeks participants attended 2 supervised exercise sessions a week, made up of 30 min of aerobic exercise. In the final 6 weeks of intervention, participants attended the university facility weekly and were advised to do home-based exercise twice a week.	Multicomponent exercise and dietary advice.	A 12-week lifestyle intervention on supervised and home-based exercise sessions and dietary advice.	A standard care (a holistic nurse-led CRC follow-up service).	Change in diet, physical activity, body weight and perception in QoL.
Gordon 2015 [37]	12 months	Health coaching was delivered over a six-month period, consisting of weekly sessions for the first five months, followed by a final telephone session conducted four weeks later.	A telephone-delivered multiple health behaviour change intervention.	Telephone-delivered sessions that addressed a specific acceptance and commitment to therapy processes about lifestyle behaviours (values, mindfulness, diffusion, acceptance and committed action, and strategies) to enhance improvement in health behaviours consistent with the Australian recommendations.	Usual care (participants were provided with four publicly available educational brochures developed by Cancer Council Australia covering CRC awareness, cancer risk reduction, nutrition, and physical activity).	Change in QoL.
Hawkes 2013 [38]	12 months	Health coaching was delivered over a six-month period, consisting of weekly sessions for the first five months, followed by a final telephone session conducted four weeks later.	A telephone-delivered multiple health behaviour change intervention.	Telephone-delivered sessions that addressed a specific acceptance and commitment to therapy processes about lifestyle behaviours (values, mindfulness, diffusion, acceptance and committed action, and strategies) to enhance improvement in health behaviours consistent with the Australian recommendations.	Usual care (participants were provided with four publicly available educational brochures developed by Cancer Council Australia covering CRC awareness, cancer risk reduction, nutrition, and physical activity).	Change in BMI, physical activity, diet, health-related quality of life (HRQOL).
Hawkes 2014 [39]	12 months	Health coaching was delivered over a six-month period, consisting of weekly sessions for the first five months, followed by a final telephone session conducted four weeks later.	A telephone-delivered multiple health behaviour change intervention.	Telephone-delivered sessions that addressed a specific acceptance and commitment to therapy processes about lifestyle behaviours (values, mindfulness, diffusion, acceptance and committed action, and strategies) to enhance improvement in health behaviours consistent with the Australian recommendations.	Usual care (participants were provided with four publicly available educational brochures developed by Cancer Council Australia covering CRC awareness, cancer risk reduction, nutrition, and physical activity).	Change in psychosocial outcomes and QoL.
Hawkes 2012 [40]	6 weeks	Health coaching for six weeks for one hour each (an introductory session followed by four weekly and one fortnightly session).	Multiple health behaviour changes interventions.	Health coaching training on moderate−vigorous physical activity, television viewing, diet, alcohol intake, BMI, and waist circumference.	Baseline measurements.	Change in health behaviours (physical activity, television viewing, diet, alcohol, and body weight).
Ho 2020 [42]	24 months	12-month interventions: individual face-to-face motivational interviewing (two sessions for Groups A and B; one session for Group C), in combination with fortnightly motivational telephone contacts, monthly mailed educational pamphlets tailored to their stage of change, quarterly newsletters, and quarterly group-based sessions.	Dietary and physical activity intervention.	Dietary, physical activity, or multicomponent intervention (received 5 general leaflets on healthy lifestyle plus the “Moving Bright, Eating Smart intervention programme”).	Usual care (received five pamphlets containing general healthy lifestyle advice, such as healthy eating, increased physical activity, weight management, smoking cessation, and limiting alcohol use, which were emailed to participants at two-month intervals over the initial 12 months). Unlike the intervention groups, the control group did not receive personalised dietary or physical activity interventions.	Change in generic and cancer-specific QoL.
Lee 2018 [43]	24 months	12-month interventions: individual face-to-face motivational interviewing (two sessions for Groups A and B; one session for Group C), in combination with fortnightly motivational telephone contacts, monthly mailed educational pamphlets tailored to their stage of change, quarterly newsletters, and quarterly group-based sessions.	Dietary and physical activity intervention.	Dietary, physical activity, or multicomponent intervention (received 5 general leaflets on healthy lifestyle plus “Moving Bright, Eating Smart intervention programme”).	Usual care (received five pamphlets containing general healthy lifestyle advice, such as healthy eating, increased physical activity, weight management, smoking cessation, and limiting alcohol use, which were emailed to participants at two-month intervals over the initial 12 months). Unlike the intervention groups, the control group did not receive personalised dietary or physical activity interventions.	Change in diet or physical activity.
Lynch 2014 [41]	12 months	Health coaching was delivered over a six-month period, consisting of weekly sessions for the first five months, followed by a final telephone session conducted four weeks later.	A telephone-delivered multiple health behaviour change intervention.	Telephone-delivered sessions that addressed a specific acceptance and commitment to therapy processes about lifestyle behaviours (values, mindfulness, diffusion, acceptance and committed action, and strategies) to enhance improvement in health behaviours consistent with the Australian recommendations.	Usual care (participants were provided with four publicly available educational brochures developed by Cancer Council Australia covering CRC awareness, cancer risk reduction, nutrition, and physical activity).	Sedentary behaviour change.
Macleod 2018 [45]	7 months	31-week interventions delivered across three phases (pre-surgery, surgical recovery, and post-treatment recovery), consisting of three face-to-face counselling sessions supplemented by nine telephone calls, all facilitated by trained lifestyle coaches.	Multiple health behaviour interventions.	Three face-to-face counselling sessions plus nine phone calls by lifestyle coaches over three phases: pre-surgery, surgical recovery and post-treatment recovery. The training programme covered smoking cessation, increasing moderate physical activity, brief interventions on alcohol and weight management.	Baseline measurements.	Changes in body weight, walking and self-reported physical activity, diet, smoking, alcohol intake, bowel function and QoL.
Yang 2020 [46]	3 months	Motivational interviewing or consultation at discharge for 30 min and at a 1-month and 3-month clinic follow-up for 15–20 min.	Multiple lifestyle interventions.	Provided with a CRC education handbook and healthy lifestyle education by an occupational therapist.	Usual care (provided with a CRC education handbook during discharge preparation but did not receive any further occupational therapy consultations.	Change in QoL and lifestyle behaviour.

Usual or standard care was defined as a routine standard treatment given by medical teams for all participants who visited hospitals for treatment. CRC: colorectal cancer; QoL: quality of life; BMI: body mass index.

**Table 3:** Effect of multicomponent lifestyle intervention on body weight changes.

Study	Outcome	Endpoint Timeframe	Intervention Group			Control Group			*p*-Value
			Baseline: Mean ± SD	Endpoint: Mean ± SD	Mean difference (MD) (95% CI)	Baseline: Mean ± SD	Endpoint: Mean ± SD	Mean difference (MD) (95% CI)	
Bourke 2011 [44]	BMI	12 weeks	26.9 ± 3.8	26.5 ± 3.3	−0.4 (−0.8 to 0.1)	27 ± 3.5	26.8 ± 3.6	−0.2 (−0.7 to 0.4)	0.429
	Waist-to-hip ratio	12 weeks	0.9 ± 0.1	0.8 ± 0.1	−0.05 (−0.1 to −0.01) *	0.9 ± 0.1	0.94 ± 0.07	0.01 (−0.06 to 0.07)	0.002
Macleod 2018 [45]	BMI (kg/m^2^): median (lower quartile, upper quartile)	7 months	28.3 (25.5, 33.5)	28.6 (26.1, 33.6)	NA	NA	NA	NA	NA
Lynch 2014 [41]	Obese (30 kg/m^2^)	6 months	NA	NA	−0.5 (−1.36 to 0.36)	NA	NA	−0.27 (−1.15 to 0.61)	NA
		12 months	NA	NA	−0.39 (−1.26 to 0.48)	NA	NA	−1.29 (−2.17 to −0.41)	NA
	Nonobese (<30 kg/m^2^)	6 months	NA	NA	−0.69 (−1.28 to −0.1) *	NA	NA	−0.47 (−1.05 to 0.1)	NA
		12 months	NA	NA	−1.42 (−2.03 to −0.81) *	NA	NA	−0.32 (−0.92 to 0.28)	NA
Hawkes 2012 [40]	Body mass index, kg/m^2^	6 weeks	30.4 ± 7.4	29 ± 6.1	−1.4 (−2.3 to −0.5) *	NA	NA	NA	<0.01
	Waist circumference, cm	6 weeks	97.5 ± 18.4	92.4 ± 15.7	−5.1 (−8.3 to −2) *	NA	NA	NA	<0.01
Hawkes 2013 [38]	BMI, kg/m^2^	6 months	24.5 ± 4.8	0.1 ± 0.2 ^#^	Adjusted between-group difference in mean change	26.4 ± 4.7	0.7 ± 0.2 ^#^	−0.5 (−1.0 to 0.0) *	0.035
		12 months	24.5 ± 4.8	0.4 ± 0.2 ^#^		26.4 ± 4.7	1.3 ± 0.2 ^#^	−0.9 (−1.4 to 0.4) *	0.001

^#^ indicates standard error. * indicates significant findings. SD—standard deviation; MD—mean difference; CI—confidence interval. NA—not applicable/reported.

**Table 4:** Effect of multicomponent lifestyle intervention on change in dietary intake.

Study	Outcome	Endpoint Timeframe	Intervention Group			Control Group			*p*-Value
			Baseline: Mean ± SD	Endpoint: Mean ± SD	Mean difference (MD) (95%CI)	Baseline: Mean ± SD	Endpoint: Mean ± SD	Mean difference (MD) (95% CI)	
Bourke 2011 [44]	Energy (kcal)	12 weeks	2012 ± 488	1755 ± 395	−257 (19 to 495)	2027 ± 348	1802 ± 361	−270 (−6–544)	0.932
	Total fat (g)		72 ± 28	60 ± 15	−12 (−35 to 11)	86 ± 17	79 ± 23	−7 (−23 to 9)	0.117
	Carbohydrate (g)		263 ± 66	227 ± 74	−36 (−64 to 7)	244 ± 55	209 ± 53	−35 (−71 to 2)	0.898
	Starch (g)		145 ± 56	123 ± 52	−22 (−41 to −4) *	135 ± 45	112 ± 35	−23 (−40 to −5)	0.814
	Fibre (g)		18 ± 7	19 ± 7	1 (−2 to 4)	16 ± 2	13 ± 4	−3 (−8 to 1)	0.044
	Saturated fat (g)		27 ± 11	23 ± 7	−4 (−12 to 5)	33 ± 9	31 ± 10	−2 (−8 to 5)	0.153
	Monounsaturated fat (g)		23 ± 9	23 ± 6	−3 (−9 to 3)	27 ± 7	25 ± 7	−2 (−11 to 6)	0.293
	Cholesterol (mg)		142 ± 65	260 ± 137	18 (−104 to 140)	291 ± 101	301 ± 94	10 (−71 to 92)	0.597
	Alcohol (g): median (range)		7 (0–28)	3 (0–22)	−4	1 (0–15)	0 (0–24)	−1	0.297
	Polyunsaturated fat (g): median (range)		12 (7–20)	10 (7–23)	−2	11 (4–34)	9 (5–15)	−2	0.310
	Sugars (g): median (range)		102 (82–163)	101 (44–135)	−1	110 (67–198	100 (37–177)	−10	0.987
Lee 2018 [43]	Red and processed meat intake (servings/week)	6 months	8.7 ± 5.8	4.3 ± 4	−2.8 (−4.0 to −1.6) *	8.2 ± 5.9	7.3 ± 5.8	NA	<0.001
		12 months	8.7 ± 5.8	3.8 ± 3.8	−3.5 (−4.6 to −2.3) *	8.2 ± 5.9	7 ± 4.7	NA	<0.001
		18 months	8.7 ± 5.8	4.5 ± 4.4	−2.5 (−3.7 to −1.3) *	8.2 ± 5.9	7.7 ± 5.7	NA	<0.001
		24 months	8.7 ± 5.8	4.6 ± 3.6	−2.9 (−3.9 to −2.0)	8.2 ± 5.9	7 ± 4.5	NA	<0.001
	Refined grain intake (servings/day)	6 months	2.8 ± 1	1.9 ± 0.8	−0.50 (−0.72 to −0.27) *	2.7 ± 0.9	2.5 ± 1.1	NA	<0.001
		12 months	2.8 ± 1	2 ± 0.9	−0.31 (−0.52 to −0.09) *	2.7 ± 0.9	2.4 ± 1	NA	0.005
		18 months	2.8 ± 1	2.1 ± 0.8	−0.38 (−0.60 to −0.17) *	2.7 ± 0.9	2.3 ± 0.9	NA	<0.001
		24 months	2.8 ± 1	2 ± 0.7	−0.45 (−0.62 to −0.29) *	2.7 ± 0.9	2.4 ± 0.8	NA	<0.001
Hawkes 2012 [40]	Red meat (serves/week)	6 weeks	2.8 ± 1.5	2.8 ± 1.5	0.02 (−0.6 to 0.6)	NA	NA	NA	0.93
	Processed meat (serves/week)	6 weeks	2 ± 1.8	0.9 ± 1.2	−1.2 (−1.8 to −0.5) *	NA	NA	NA	<0.01
	Fruit (serves/day)	6 weeks	1.8 ± 0.9	2.2 ± 1.2	0.3 (−0.3 to 0.9)	NA	NA	NA	0.27
	Vegetables (serves/day)	6 weeks	2.5 ± 1.3	3.5 ± 1.1	1 (0.4 to 1.6)	NA	NA	NA	<0.01
Hawkes 2013 [38]	Total fat, % of kJ intake	6 months	64.2 ± 29.9	−2.1 ± 1.8 ^#^	−8.5 (−13.4 to 3.6)	64.7 ± 29.6	6.5 ± 1.7 ^#^	Adjusted between-group difference in mean change	0.001
		12 months	64.2 ± 29.9	−0.6 ± 1.8 ^#^	−7.0 (−12.0 to 2.0)	64.7 ± 29.6	6.4 ± 1.8 ^#^		0.006
	Saturated fat, % of kJ intake	6 months	25.9 ± 13.7	−1.7 ± 0.8 ^#^	−3.5 (−5.7 to 1.2)	26.4 ± 13.8	1.8 ± 0.8 ^#^		0.002
		12 months	25.9 ± 13.7	−0.8 ± 0.8 ^#^	−2.8 (−5.1 to 0.5	26.4 ± 13.8	2.0 ± 0.8 ^#^		0.016
	Fibre, g per day	6 months	20.7 ± 8.3	0.3 ± 0.6 ^#^	−0.7 (−2.2 to 0.8)	20.4 ± 7.9	1.0 ± 0.5 ^#^		0.383
		12 months	20.7 ± 8.3	−0.2 ± 0.6 ^#^	−1.0 (−2.5 to 0.6)	20.4 ± 7.9	0.7 ± 0.6 ^#^		0.223
	Fruit, servings per day	6 months	1.8 ± 1.1	0.4 ± 0.1 ^#^	−0.2 (−0.0 to 0.4)	1.8 ± 1.2	0.2 ± 0.1 ^#^		0.119
		12 months	1.8 ± 1.1	0.2 ± 0.1 ^#^	0.0 (−0.2 to 0.3)	1.8 ± 1.2	0.2 ± 0.1 ^#^		0.751
	Vegetables, servings per day	6 months	3.6 ± 1.2	0.3 ± 0.1 ^#^	−0.4 (−0.2 to 0.7)	3.6 ± 1.3	−0.1 ± 0.1 ^#^		0.001
		12 months	3.6 ± 1.2	0.2 ± 0.1 ^#^	0.2 (0.1 to 0.5)	3.6 ± 1.3	0.0 ± 0.1 ^#^		0.139
	Alcohol, g per day	6 months	4.6 ± 10.6	3.6 ± 0.9 ^#^	−1.4 (−3.7 to 1.0)	6.2 ± 13.8	4.9 ± 0.8 ^#^		0.259
		12 months	4.6 ± 10.6	4.4 ± 0.9 ^#^	−0.6 (−3.0 to 1.8)	6.2 ± 13.8	5.1 ± 0.9 ^#^		0.607

^#^ Indicates standard error. * indicates significant findings. SD—standard deviation; MD—mean difference; CI—confidence interval; NA—not applicable/reported.

**Table 5:** Effect of multicomponent lifestyle intervention on change in physical activities.

Study	Outcome	Endpoint Timeframe	Intervention Group			Control Group			*p*-Value
			Baseline: Mean (SD)	Endpoint: Mean (SD)	Mean difference (MD); 95%CI	Baseline: Mean (SD)	Endpoint: Mean (SD)	Mean difference (MD); 95%CI	
Bourke 2011 [44]	Maximum voluntary torque (Nm)	12 weeks	187.9 ± 27.1	189.2 ± 27.7	1.3 (−5 to 7.7)	153.1 ± 37.5	169 ± 45.6	15.9 (−1.7 to 33.4)	0.127
	Exercise tolerance (s)	12 weeks	404.2 ± 113.1	528.2 ± 114.5	199.9 (75.4 to 164.4) *	328.3 ± 120	376.7 ± 125.7	48.4 (18.4 to 81.3)	0.01
Hawkes 2013 [38]	Moderate	6 months	37.3 ± 88.4	59.2 ± 128	16.5 (−7.4 to 40.5)	42.2 ± 96.2	47.2 ± 100.5	Adjusted between-group difference in mean change	0.176
		12 months	37.3 ± 88.4	59.1 ± 142.4	28.5 (3.9 to 53.1) *	42.2 ± 96.2	34.8 ± 80.8		0.023
	Vigorous	6 months	10.8 ± 45.7	13 ± 74.3	−2.7 (3.9 to 53.1)	4.9 ± 29.4	9.9 ± 49.2		0.023
		12 months	10.8 ± 45.7	13 ± 49.5	−2.5 (−14.4 to 9.3)	4.9 ± 29.4	9.7 ± 45.4		0.676
	Moderate-to-vigorous physical activity	6 months	58.9 ± 132.9	85.1 ± 197.9	11.5 (−18.8 to 41.9)	52 ± 112.5	66.7 ± 139.2		0.457
		12 months	58.9 ± 132.9	85.2 ± 181	23.7 (−7.4 to 54.8)	52 ± 112.5	54.3 ± 120		0.136
Lee 2018 [43]	Physical activity: level (accumulated minutes/week of moderate-to-vigorous intensity physical activity)	6 months	498.2 ± 289.8	660.6 ± 317.5	21.6 (−61 to 104.3)	485.1 ± 290.7	612.4 ± 325.8	NA	0.607
		12 months	498.2 ± 289.8	594.7 ± 238.3	10.1 (−78.2 to 98.3)	485.1 ± 290.7	578.4 ± 291.4	NA	0.823
		18 months	498.2 ± 289.8	681.8 ± 309.7	3.3 (−81.7 to 88.3)	485.1 ± 290.7	643.8 ± 291.4	NA	0.939
		24 months	498.2 ± 289.8	705 ± 324	73.1 (−12.5 to 158.8)	485.1 ± 290.7	613.3 ± 321.4	NA	0.094
Lynch 2015 [41]	Sufficient activity (150 min/wk.)	6 months	NA	NA	−0.94 (−2.03 to 0.15)	NA	NA	0.77 (−0.37–1.92)	NA
		12 months	NA	NA	−0.99 (−2.12 to 0.15)	NA	NA	−0.15 (−1.43 to 1.14)	NA
	Insufficient activity (<150 min/wk).	6 months	NA	NA	−0.63 (−1.19 to −0.07) *	NA	NA	−0.62 (−1.17 to −0.08)	NA
		12 months	NA	NA	−21.25 (−21.82 to −20.67) *	NA	NA	−20.62 (−21.18 to −20.07)	NA
	Watching television/videos (h/d)	6 months	NA	NA	−0.36 (−0.59 to −0.15) *	NA	NA	−0.22 (−0.43 to 0)	NA
		12 months	NA	NA	−0.49 (−0.71 to −0.26) *	NA	NA	−0.29 (−0.52 to −0.07)	NA
	Screen time (h/d)	6 months	NA	NA	−0.41 (−0.67 to −0.16) *	NA	NA	−0.17 (−0.43 to 0.07)	NA
		12 months	NA	NA	−0.56 (−0.83 to −0.3) *	NA	NA	−0.24 (−0.5 to 0.02)	NA
	Total sedentary time (h/d)	6 months	NA	NA	−0.65 (−1.14 to −0.15) *	NA	NA	−0.44 (−0.93 to 0.05)	NA
		12 months	NA	NA	−1.21 (−1.71 to −0.7) *	NA	NA	−0.55 (−1.06 to −0.05)	NA
Hawkes 2012 [40]	Mean change in moderate-to-vigorous physical activity	6 weeks	66.6 ± 21.1	217.3 ± 34.7	150.7 (22.7 to 110.5) *	NA	NA	NA	<0.01
	TV viewing, h/week	6 weeks	12.9 ± 1.7	11.5 ± 1.6	−1.4 (−4 to 1.2)	NA	NA	NA	0.28

* Significant findings. SD—standard deviation; MD—mean difference; CI—confidence interval; NA—not applicable/reported.

**Table 6:** Effect of multicomponent lifestyle intervention on quality of life.

Study	Outcome/Validated Tool Used to Measure QoL	Endpoint Timeframe	Intervention Group			Control Group			
			Baseline: Mean (SD)	Endpoint: Mean (SD)	Mean difference (MD); 95%CI	Baseline: Mean (SD)	Endpoint: Mean (SD)	Mean difference (MD); 95%CI	
Bourke 2011 [44]	FACT-C (functional assessment of cancer therapy—colorectal)	12 weeks	120 ± 10	120 ± 11	0 (−3 to 3)	102 ± 15	106 ± 13	4 (−5 to 12)	0.795
	FACT-F (functional assessment of cancer therapy—fatigue)	12 weeks	43 ± 7	48 ± 4	5 (1 to 8) *	42 ± 9	43 ± 6	1 (−1 to 4)	0.005
Ho 2020 [42]	QoL: SF-6D utility index (0–1)	6 months	0.8 ± 0.2	0.9 ± 0.1	NA	0.8 ± 0.2	0.9 ± 0.2	Mean difference was presented for only the diet or physical activity intervention, but not for the multicomponent one	NA
		12 months	0.8 ± 0.2	0.9 ± 0.1	NA	0.8 ± 0.2	0.9 ± 0.2		NA
		18 months	0.8 ± 0.16	0.9 ± 0.1	NA	0.8 ± 0.2	0.9 ± 0.1		NA
		24 months	0.8 ± 0.2	0.9 ± 0.1	NA	0.8 ± 0.2	0.9 ± 0.1		NA
	QoL: SF-12 PCS (0–100)	6 months	46.7 ± 7.1	50.7 ± 5.4	NA	46.4 ± 6.7	47.8 ± 7.2		NA
		12 months	46.7 ± 7.1	50.1 ± 5.1	NA	46.4 ± 6.7	47.8 ± 6.8		NA
		18 months	46.7 ± 7.1	50.1 ± 6.2	NA	46.4 ± 6.7	48 ± 7.8		NA
		24 months	46.7 ± 7.1	51.5 ± 5.1	NA	46.4 ± 6.7	48.4 ± 7.5		NA
	QoL: SF-12 MCS (0–100)	6 months	53.2 ± 8.5	57.8 ± 6.4	NA	50.2 ± 10.6	56 ± 8.8		NA
		12 months	53.2 ± 8.5	59.5 ± 4.5	NA	50.2 ± 10.6	58 ± 7.3		NA
		18 months	53.2 ± 8.5	58.6 ± 6.7	NA	50.2 ± 10.6	57.1 ± 8.6		NA
		24 months	53.2 ± 8.5	59.9 ± 4.3	NA	50.2 ± 10.6	60.1 ± 5.5		NA
	QoL: FACT-C total score (0–132)	6 months	110.4 ± 18.2	122.7 ± 14.2	NA	106.9 ± 16.6	120.6 ± 16.8		NA
		12 months	110.4 ± 18.2	126.6 ± 9.6	NA	106.9 ± 16.6	120.3 ± 15.5		NA
		18 months	110.4 ± 18.2	123.7 ± 13.5	NA	106.9 ± 16.6	121.3 ± 17.3		NA
		24 months	110.4 ± 18.2	127.9 ± 11	NA	106.9 ± 16.6	125.1 ± 14.6		NA
	QoL: FACT-G total score (0–104)	6 months	82.8 ± 13.5	91.7 ± 11	NA	79.1 ± 13.8	90 ± 13.3		NA
		12 months	82.8 ± 13.5	95.8 ± 7.6	NA	79.1 ± 13.8	90.2 ± 12.3		NA
		18 months	82.8 ± 13.5	92.5 ± 10.5	NA	79.1 ± 13.8	90.9 ± 13.8		NA
		24 months	82.8 ± 13.5	96.4 ± 8.3	NA	79.1 ± 13.8	93.9 ± 11.7		NA
	HADS—anxiety (0–21)	6 months	10.6 ± 4	8.9 ± 2.4	NA	10.7 ± 3.8	9 ± 3.2		NA
		12 months	10.6 ± 4	8.4 ± 1.6	NA	10.7 ± 3.8	9.1 ± 3.1		NA
		18 months	10.6 ± 4	8.6 ± 2.2	NA	10.7 ± 3.8	9 ± 3.3		NA
		24 months	10.6 ± 4	8 ± 1.7	NA	10.7 ± 3.8	8.4 ± 2.7		NA
	HADS—depression (0–21)	6 months	11.9 ± 3.7	10.8 ± 3.4	NA	11.8 ± 3.4	11.3 ± 3.2		NA
		12 months	11.9 ± 3.7	9.3 ± 2.2	NA	11.8 ± 3.4	10.7 ± 2.8		NA
		18 months	11.9 ± 3.7	9.6 ± 2.6	NA	11.8 ± 3.4	10.7 ± 3.6		NA
		24 months	11.9 ± 3.7	8.5 ± 1.8	NA	11.8 ± 3.4	10.1 ± 3		NA
Hawkes 2013 [38]	SF-36 HRQoL (0–100), physical component summary	6 months	44.6 ± 10.7	3.4 ± 0.6 ^#^	−0.0 (−1.8 to 1.8)	45.5 ± 9.1	3.4 ± 0.6 ^#^	Adjusted between-group difference in mean change	0.991
		12 months	44.6 ± 10.7	4.8 ± 0.7 ^#^	1.7 (−0.2 to 3.5)	45.5 ± 9.1	3.2 ± 0.7 ^#^		0.072
	SF-36 HRQoL (0–100), mental component summary	6 months	50.2 ± 10.1	1.9 ± 0.6 ^#^	0.7 (−1.1 to 2.5)	50.9 ± 9.5	1.2 ± 0.6 ^#^		0.455
		12 months	50.2 ± 10.1	0.3 ± 0.7 ^#^	−0.7 (−2.5 to 1.2)	50.9 ± 9.5	1 ± 0.7 ^#^		0.472
Gordon 2015 [37]	SF-6D	6 months	0.7 ± 0.1	0.7 ± 0.1	0.002 (−0.020 to 0.024)	0.7 ± 0.1	0.8 ± 0.1	Which mean difference is not explained and probably may be adjusted between-group difference in mean change	NA
		12 months	0.7 ± 0.1	0.75 ± 0.1	0.004 (−0.019 to 0.028)	0.7 ± 0.1	0.8 ± 0.1		NA
	SF-36 physical component	6 months	44.6 ± 10.7	48.5 ± 10.4	−0.0 (−1.8 to 1.8)	45.5 ± 9.2	49.2 ± 9.2		NA
		12 months	44.6 ± 10.7	50.1 ± 9.5	1.7 (−0.2 to 3.5)	45.5 ± 9.2	49.3 ± 8.9		NA
	SF-36 mental component	6 months	50.2 ± 10.1	52.3 ± 8.5	0.7 (−1.1 to 2.5)	50.9 ± 9.5	52.7 ± 8		NA
		12 months	50.2 ± 10.1	50.7 ± 9.1	−0.7 (−2.5 to 1.2)	50.9 ± 9.5	52.4 ± 8.1		NA
Hawkes 2014 [39]	FACT-C version 4 cancer-specific quality of life (range 0–132)	6 months	109.8 ± 16.5	5.4 ± 0.9 ^#^	1.9 (−0.6 to 4.3)	112.7 ± 112.7	3.6 ± 0.9 ^#^	Adjusted between-group difference in mean change	NA
		12 months	109.8 ± 16.5	6 ± 0.9 ^#^	1.8 (−0.7 to 4.3)	112.7 ± 112.7	4.2 ± 0.9 ^#^		NA
	Cancer-specific quality of life—physical well-being (0–28)	6 months	22.9 ± 5.2	1.8 ± 0.3 ^#^	0.8 (0 to 1.6)	24.1 ± 24.1	1 ± 0.3 ^#^		NA
		12 months	22.9 ± 5.2	2.2 ± 0.3 ^#^	0.9 (0.1 to 1.7)	24.1 ± 24.1	1.3 ± 0.3 ^#^		NA
	Cancer-specific quality of life—social well-being (0–28)	6 months	22.6 ± 4.8	0.9 ± 0.3 ^#^	0.1 (−0.7 to 0.9)	22.9 ± 5.2	0.7 ± 0.3 ^#^		NA
		12 months	22.6 ± 4.8	0.3 ± 0.3 ^#^	−0.3 (−1.1 to 0.6)	22.9 ± 5.2	0.5 ± 0.3 ^#^		NA
	Cancer-specific quality of life—emotional well-being (0–24)	6 months	20.9 ± 3	0.5 ± 0.2 ^#^	0.3 (−0.3 to 0.8)	21.2 ± 3	0.5 ± 0.2 ^#^		NA
		12 months	20.9 ± 3	0.4 ± 0.2 ^#^	0.3 (−0.2 to 0.8)	21.2 ± 3	0.4 ± 0.2 ^#^		NA
	Cancer-specific quality of life—functional well-being (0–28)	6 months	21.4 ± 5.2	0.8 ± 0.3 ^#^	0.4 (−0.4 to 1.3)	22.4 ± 4.6	0.8 ± 0.3 ^#^		NA
		12 months	21.4 ± 5.2	1.1 ± 0.3 ^#^	0.5 (−0.4 to 1.3)	22.4 ± 4.6	1.1 ± 0.3 ^#^		NA
	Cancer-specific quality of life—additional well-being (0–36)	6 months	22.1 ± 3.9	0.7 ± 0.3 ^#^	0.2 (−0.6 to 0.9)	22 ± 4.1	0.7 ± 0.3 ^#^		NA
		12 months	22.1 ± 3.9	1.0 ± 0.3 ^#^	0.3 (−0.4 to 1.1)	22 ± 4.1	1 ± 0.3 ^#^		NA
	Cancer-specific quality of life—trial outcome index	6 months	66.3 ± 11.9	2.4 ± 0.7 ^#^	1.4 (−0.5 to 3.4)	68.5 ± 10.8	2.4 ± 0.7 ^#^		NA
		12 months	66.3 ± 11.9	3.3 ± 0.7 ^#^	1.7 (−0.2 to 3.7)	68.5 ± 10.8	3.3 ± 0.7 ^#^		NA
Hawkes 2012 [40]	SF-36 Health-related quality of life (HRQoL)								
	Physical functioning	6 weeks	50.6 ± 6.5	52.8 ± 4.4	2.2 (0.9 to 3.5)	NA	NA	NA	<0.01
	Role physical	6 weeks	47.2 ± 8.5	50.2 ± 7.7	3.1 (−0.9 to 7)	NA	NA	NA	0.12
	Bodily pain	6 weeks	45 ± 11.4	50.7 ± 9.7	5.8 (1.4 to 10.1) *	NA	NA	NA	0.01
	General health	6 weeks	48.4 ± 29.6	52.1 ± 8.6	3.7 (0.3 to 7.1)	NA	NA	NA	0.03
	Vitality	6 weeks	47.3 ± 42.7	52 ± 49.3	4.8 (1.4 to 8.2) *	NA	NA	NA	0.01
	Social functioning	6 weeks	48 ± 43.2	52.3 ± 49.8	4.3 (−0.4 to 9)	NA	NA	NA	0.07
	Role emotional	6 weeks	47 ± 14.2	50.7 ± 6.5	3.7 (−2 to 9.4)	NA	NA	NA	0.19
	Mental health	6 weeks	45.8 ± 13.7	50.6 ± 6.8	4.8 (−0.7 to 10.3)	NA	NA	NA	0.08
	Physical HRQoL	6 weeks	48.6 ± 1.6	51.9 ± 6.6	3.3 (0.3 to 6.3)	NA	NA	NA	0.03
	Mental HRQoL	6 weeks	46.4 ± 14.9	50.8 ± 6.9	4.4 (−1.9 to 10.7)	NA	NA	NA	0.16
Yang 2020 [46]	Taiwan version of the WHOOQOL-BREF physical health	1 month	12 ± 1.7	14.5 ± 1.9	NA	12.3 ± 1.9	14.1 ± 1.9	NA	NA
		3 months	12 ± 1.7	15.3 ± 1.7	NA	12.3 ± 1.9	14.6 ± 1.7	NA	NA
	Psychological health	1 month	12.8 ± 2.4	13.7 ± 2.5	NA	12.2 ± 1.9	13.2 ± 2	NA	NA
		3 months	12.8 ± 2.4	14.2 ± 2.2	NA	12.2 ± 1.9	13.6 ± 1.8	NA	NA
	Social relationships	1 month	13.8 ± 2.1	13.7 ± 1.9	NA	13.2 ± 1.7	13.6 ± 1.7	NA	NA
		3 months	13.8 ± 2.1	14.5 ± 2.1	NA	13.2 ± 1.7	13.7 ± 1.6	NA	NA
	Environment	1 month	13.7 ± 2.1	14.3 ± 2.1	NA	13.4 ± 1.6	14.3 ± 1.9	NA	NA
		3 months	13.7 ± 2.1	14.5 ± 2	NA	13.4 ± 1.6	14.4 ± 1.6	NA	NA
	Barthel index	1 month	80.9 ± 14.7	96 ± 11.7	NA	77.2 ± 17.3	97.2 ± 6.3	NA	NA
		3 months	80.9 ± 14.7	99 ± 3.3	NA	77.2 ± 17.3	98.3 ± 5.6	NA	NA
	Handbook self-checklist score	1 month	80.9 ± 14.7	53.5 ± 8.7	NA	77.2 ± 17.3	51.9 ± 7.1	NA	NA
		3 months	80.9 ± 14.7	58.1 ± 10.5	NA	77.2 ± 17.3	53.2 ± 5.5	NA	NA
	Brief Symptom Rating Scale (BSRS-5)	1 month	5.3 ± 3.1	3 ± 2.8	NA	5.2 ± 3.1	2.7 ± 2.1	NA	NA
		3 months	5.3 ± 3.1	1.8 ± 2.3	NA	5.2 ± 3.1	1.9 ± 2.5	NA	NA

^#^ indicates standard error. * indicates significant findings. SF-6D = six-dimensional health state short form; SF-12PCS = Physical Component Score of 12-Item Short Form Health Survey; SF-12MCS = Mental Component Score of 12-Item Short Form Health Survey; FACT-C = Functional Assessment of Cancer Therapy—Colorectal Scale; FACT-G = Functional Assessment of Cancer Therapy—general score; HADS = Hospital Anxiety and Depression Scale; WHOOQOL-BREF World Health Organisation Quality of Life Brief; SD—standard deviation; MD—mean difference; CI—confidence interval; NA—not applicable/reported.

## Data Availability

All data are presented in the manuscript.

## Supplementary Materials

The following supporting information can be downloaded at: https://www.mdpi.com/article/10.3390/cancers18121906/s1; Table S1: Search strategy for Medline/Ovid (search last updated on 11 April 2025). Table S2: Reasons for exclusion of studies after full text reading. Table S3: Characteristics of cases lost to follow-up and funding information of the included studies.
