# Supplementary material for: Multicomponent Lifestyle Interventions During Colorectal Cancer Surveillance: A Systematic Review
Source: Cancers (Basel). 2026 Jun 11;18(12):1906. doi: 10.3390/cancers18121906 (PMC13297519; doi:10.3390/cancers18121906)
Supplement: Supplementary file 1 [file cancers-18-01906-s001.zip › cancers-4307137-supplementary.pdf]

Supplementary material

Supplementary Table S1: Search strategy for Medline/Ovid (search last updated on April 11, 2025).

#	Searches	Results
1	colorectal neoplasms/ or colonic neoplasms/ or rectal neoplasms/	237636
2	((colorect* or colo* or rect* or bowel) adj3 (neoplas* or cancer* or tumor* or tumour* or carcinoma* or adenocarcinoma* or malignan*)).tw,kf.	310609
3	1 or 2	355418
4	overweight/ or obesity/ or obesity, abdominal/ or body mass index/ or waist-hip ratio/	351181
5	(overweight or obesity).tw,kf	401185
6	((weight or "BMI" or "body mass index") adj5 (presser* or maintain* or management or reduc* or los* or decreas* or control)).tw,kf.	255061
7	Exercise/ or Physical Fitness/	178449
8	Exercise Therapy/ or Physical Therapy Modalities/	93747
9	("mvpa" or "moderate to vigorous physical activity" or "physical activit*" or exercis* or physiotherapy).tw,kf.	558081
10	((Fitness or exercise) adj3 (lack or inactivity or training or aerobic or isometric)).tw,kf.	50643
11	exp diet/ or energy intake/ or portion size/ or serving size/	352250
12	Feeding Behavior/	97546
13	Eating/ and Behavior/	120
14	((food or calorie or nutrition* or eating or diet* or energy or feeding) adj5 (intake or portion or size or pattern* or habit or habits or factor* or behavi* or intervention*)).tw,kf.	358898
15	alcohol drinking/ or binge drinking/	80687
16	(alcohol adj3 (drink* or consum* or intak* or habit* or behavi* or excess*)).tw,kf.	99218
17	Cigarette Smoking/ or Smoking Cessation/ or Tobacco Smoking/ or Smoking/ or Smoking Reduction/ or Smoking Pipes/ or Electronic Nicotine Delivery Systems/ or Vaping/	180144
18	((smoke or smoking or cigarette* or cigar* or tobacco or "electronic nicotine delivery system*" or "e-cig*" or "e-cigarettes" or vaping) adj3 (cessation or behavi* or quit* or halting* or stop* or reduc* or cease*)).tw,kf.	65921
19	life style/ or healthy lifestyle/ or life change events/ or sedentary behavior/	108263
20	(sedentary adj1 (lifestyle* or time or behav*)).tw,kf.	21069

21	(lifestyle adj3 (change* or behavio* or modified or intervention*)).tw,kf.	38193
22	or/4-21	2052741
23	"Quality of Life"/	303383
24	Health Status/	93550
25	("quality of life" or "Qol" or "HRQOL" or "hrql").tw,kf.	451541
26	(Health adj3 (status or level or general or overall)).tw,kf.	179898
27	Patient Reported Outcome Measures/	18102
28	("patient reported outcome*" or "PROMs").tw,kf.	49495
29	colorectal neoplasms/ or colonic neoplasms/ or rectal neoplasms/	237636
30	Survivors/	32526
31	29 and 30	745
32	"cancer survivor*".tw,kf.	29430
33	23 or 24 or 25 or 26 or 27 or 28 or 31 or 32	765585
34	3 and 22 and 33	1580
35	limit 34 to english language	1510

/ represents subject headings. Keywords search: search field used .tw,kf represents Title, abstract and author keywords. Keywords search: proximity search used adj with a number. Words have to be within a certain number adj5 represented within four words, as Medline adds one for space.

Supplementary Table S2: Reasons for exclusion of studies after full text reading

First author & year	Title	Reason
Anderson 2018	Feasibility study to assess the impact of a lifestyle intervention ('LivingWELL') in people having an assessment of their family history of colorectal or breast cancer	Wrong patient population
Beresford 2006	Low-fat dietary pattern and risk of colorectal cancer: the Women's Health Initiative Randomised Controlled Dietary Modification Trial	Wrong intervention
Berstad 2015	Long-term lifestyle changes after colorectal cancer screening: randomised controlled trial	Wrong patient population
Beumeler 2018	Evaluation of a lifestyle intervention program in primary care on physical and mental health and quality of life of cancer survivors: A pilot study	Wrong patient population
Campbell 2009	A randomised trial of tailoring and motivational interviewing to promote fruit and vegetable consumption for cancer prevention and control	Wrong intervention
Cantwell 2024	A two-arm non-randomised trial of MedEx IMPACT: a community-based, physical activity behaviour change intervention for survivors of cancer	Wrong intervention
Chang 2025	Effects of integrative telehealth-based nutrition care with and without oral nutritional supplements in patients with liver and colorectal cancer: A randomised controlled trial	Wrong intervention
Chebet 2020	Association of diet quality and physical activity on obesity-related cancer risk and mortality in black women: results from the Women's Health Initiative	Wrong study design
Chi 2020	Effects of Lifestyle Intervention Based on Information Technology on Healthy Outcome of Patients with Colorectal Cancer	Protocol
Chi 2021	The construction and application of a mHealth-based personalised exercise and dietary intervention for colorectal cancer survivors with fatigue	Protocol
Chi 2023	Effects and mechanism of exercise intervention on clinical outcomes in patients with advanced colorectal cancer: a randomised controlled trial	Protocol
Corle 2001	Self-rated quality of life measures: effect of change to a low-fat, high-fiber, fruit and vegetable enriched diet	Wrong intervention
Crane 2021	Lifestyle intervention for Latina cancer survivors and caregivers: the Nuestra Salud randomised pilot trial	Wrong patient population
Dimeo 2004	Effect of aerobic exercise and relaxation training on fatigue and physical performance of cancer patients after surgery.: A randomised controlled trial	Wrong intervention

Domoslawska-Zylinska 2025	Smoking Avoidance, Physical Activity and Diet as Preventative Behaviours for Lung, Prostate and Colorectal Cancer - A Comparison of the Extended Parallel Process Model Groups	Wrong study design
Grimmett 2015	Diet and physical activity intervention in colorectal cancer survivors: a feasibility study	Wrong intervention
Hawkes 2009	A telephone-delivered lifestyle intervention for colorectal cancer survivors 'CanChange': a pilot study	Wrong intervention
Hawkes 2009	Developing and pilot testing a telephone-delivered lifestyle intervention for colorectal cancer survivors - 'Canchange'	Abstract
Hawkes 2015	Predictors of physical activity in colorectal cancer survivors after participation in a telephone-delivered multiple health behavior change intervention	Wrong intervention
Hawkes 2010	'Canchange': a trial of a telephone-delivered lifestyle intervention for colorectal cancer (CRC) survivors	Conference abstract
Ibfelt 2011	No change in health behavior, BMI or self-rated health after a psychosocial cancer rehabilitation: Results of a randomised trial	Wrong intervention
ISRCTN24901641 2012	At cancer diagnosis? A? window of opportunity? for behavioural change towards physical activity for colon and breast cancer patients	Wrong intervention
Kenzik 2015	Symptoms, weight loss, and physical function in a lifestyle intervention study of older cancer survivors	Wrong patient population
Keskin 2021	Colorectal cancer in the Linxian China Nutrition Intervention Trial: Risk factors and intervention results	Wrong intervention
Land 2014	Cigarette smoking, physical activity, and alcohol consumption as predictors of cancer incidence among women at high risk of breast cancer in the NSABP P-1 trial	Wrong study design
Lanza 2007	The polyp prevention trial continued follow-up study: no effect of a low-fat, high-fiber, high-fruit, and -vegetable diet on adenoma recurrence eight years after randomisation	Wrong intervention
Morey 2009	Effects of home-based diet and exercise on functional outcomes among older, overweight long-term cancer survivors: RENEW: a randomised controlled trial	Wrong outcomes
NCT01708824 2012	Diet and Physical Activity Intervention in CRC Survivors	Protocol
NCT02724306 2016	Physical Activity Intervention With People at Increased Risk of Developing Colon Cancer	Protocol
Reedy 2005	The influence of health behavior clusters on dietary change	Wrong study design

Sansbury 2009	The effect of strict adherence to a high-fiber, high-fruit and -vegetable, and low-fat eating pattern on adenoma recurrence	Wrong intervention
Sremanakova 2023	Healthy eating and active lifestyle after bowel cancer: feasibility of a randomised controlled trial	Conference abstract
Storck 2020	Effect of a leucine-rich supplement in combination with nutrition and physical exercise in advanced cancer patients: A randomised controlled intervention trial	Wrong patient population
Sun 2025	The Relationship Between Dietary and Supplemental omega-3 Highly Unsaturated Fatty Acid Intake, Blood and Tissue omega-3 Highly Unsaturated Fatty Acid Concentrations, and Colorectal Polyp Recurrence: A Secondary Analysis of the seAFOod Polyp Prevention Trial	Wrong intervention
VanBlarigan 2018	Association of Survival With Adherence to the American Cancer Society Nutrition and Physical Activity Guidelines for Cancer Survivors After Colon Cancer Diagnosis: The CALGB 89803/Alliance Trial	Wrong study design
van der Werf 2019	The effect of nutritional counselling on muscle mass and treatment outcomes in patients with metastatic colorectal cancer undergoing chemotherapy: a randomised controlled trial	Conference abstract
Velho 2020	Adherence to a combined exercise and dietary intervention in patients with gastrointestinal cancer undergoing neo-adjuvant therapy	Conference abstract
Wang 2023	Quality of life among colorectal cancer survivors participating in a pilot randomised controlled trial of a web-based dietary intervention with text messages	Wrong intervention
Wang 2022	Personalised nutrition intervention improves nutritional status and quality of life of colorectal cancer survivors in the community: A randomised controlled trial	Wrong intervention
Winger 2014	Diet and exercise intervention adherence and health-related outcomes among older long-term breast, prostate, and colorectal cancer survivors	Wrong patient population
Wu 2020	Impact of oral nutritional supplements in post-discharge patients at nutritional risk following colorectal cancer surgery: a randomised clinical trial	Conference abstract

Supplementary Table S3: Characteristics of cases lost to follow-up and funding information of the included studies.

Study	Number of lost to follow-up and reasons	Sponsorship
Bourke 2011 (1)	1 participant was excluded from the intervention group due to a stroke.	Sheffield Hallam University
Gordon 2015 (2)	**Intervention:** At baseline: 16 did not receive intervention; at 6 months: 34 lost to follow-up, 6 unable to contact, 3 deceased, 3 too ill, 19 refused, and 3 passive refusals; at 12 months: 12 lost to follow-up, 2 unable to contact, 1 deceased, 1 too ill, 6 refused, and 2 passive refusals. **Control:** At 6 months: 29 lost to follow-up, 5 unable to contact, 3 deceased, 1 too ill, 18 refused, and 2 passive refusals; at 12 months: 13 lost to follow-up, 2 unable to contact, 2 deceased, 3 too ill, 5 refused, and 1 passive refusal.	Australian Government through Cancer Australia
Hawkes 2013 (3)	**Intervention:** At baseline: 16 did not receive intervention; at 6 months: 34 lost to follow-up, 6 unable to contact, 3 deceased, 3 too ill, 19 refused, and 3 passive refusals; at 12 months: 12 lost to follow-up, 2 unable to contact, 1 deceased, 1 too ill, 6 refused, and 2 passive refusals. **Control:** At 6 months: 29 lost to follow-up, 5 unable to contact, 3 deceased, 1 too ill, 18 refused, and 2 passive refusals; at 12 months: 13 lost to follow-up, 2 unable to contact, 2 deceased, 3 too ill, 5 refused, and 1 passive refusal.	Australian Government through Cancer Australia
Hawkes 2014 (4)	**Intervention:** At baseline: 16 did not receive intervention; at 6 months: 34 lost to follow-up, 6 unable to contact, 3 deceased, 3 too ill, 19 refused, and 3 passive refusals; at 12 months: 12 lost to follow-up, 2 unable to contact, 1 deceased, 1	Australian Government through Cancer Australia

	too ill, 6 refused, and 2 passive refusals.<br>**Control:**<br>At 6 months: 29 lost to follow-up, 5 unable to contact, 3 deceased, 1 too ill, 18 refused, and 2 passive refusals; at 12 months: 13 lost to follow-up, 2 unable to contact, 2 deceased, 3 too ill, 5 refusal, and 1 passive refusal.	
Hawkes 2012 (5)	None of the participants were lost to follow-up.	The Cancer Council Queensland
Ho 2020 (6)	**Intervention:**<br>At 6 months: 1 recurrence, 1 new cancer; at 12 months: 1 recurrence; at 18 months: 1 recurrence, 1 new cancer, 1 loss to follow-up; at 24 months: 1 recurrence, 1 loss to follow-up<br>**Controls:**<br>**Dietary only:** At 6 months: 2 recurrences, 1 new cancer, and 1 death; at 12 months: 1 recurrence, 1 death, and 2 lost to follow-up; at 18 months: 1 recurrence; at 24 months: 1 lost to follow-up<br>**Physical activity only:** At 6 months: 2 lost to follow-up; at 12 months: 1 recurrence; at 24 months:1= recurrence<br>**Usual care only:** At 6 months: 2 recurrences, 2 deaths; at 12 months: 1 recurrence, 1 death, and 2 lost to follow-up; at 18 months: 1 recurrence, 1 new cancer, and 1 loss to follow up; at 24 months: 2 recurrences.	World Cancer Research Fund International
Lee 2018 (7)	**Intervention:**<br>At 6 months: 1 recurrence, 1 new cancer; at 12 months: 1 recurrence; at 18 months: 1 recurrence, 1 new cancer, and 1 lost to follow-up; at 24 months: 1 recurrence, 1 lost to follow-up<br>**Controls:**<br>**Dietary only:** At 6 months: 2 recurrences, 1 new cancer, and 1 death; at 12 months: 1 recurrence, 1 death, 2 lost to	The World Cancer Research Fund International & the Wereld Kanker Onderzoek Fonds

	follow-up; at 18 months: 1 recurrence; at 24 months: 1 lost to follow-up.<br>**Physical activity only:** At 6 months: 2 lost to follow-up; at 12 months: 1 recurrence; at 24 months: 1= recurrence<br>**Usual care only:** At 6 months: 2 recurrences, 2 deaths; at 12 months: 1 recurrence, 1 death, and 2 lost to follow-up; at 18 months: 1 recurrence,1 new cancer, and 1 lost to follow-up; at 24 months: 2 recurrences.	
Lynch 2014 (8)	**Intervention:**<br>At baseline: 16 did not receive intervention; at 6 months: 34 lost to follow-up, 6 unable to contact, 3 deceased, 3 too ill, 19 refused, and 3 passive refusals; at 12 months: 12 lost to follow up, 2 unable to contact, 1 deceased, 1 too ill, 6 refused, 2 passive refusals.<br>**Control:**<br>At 6 months: 29 lost to follow-up, 5 unable to contact, 3 deceased, 1 too ill, 18 refused, and 2 passive refusals; at 12 months: 13 lost to follow-up, 2 unable to contact, 2 deceased, 3 too ill, 5 refusal, and 1 passive refusal.	The Australian government through Cancer Australia
Macleod 2018 (9)	7, mainly due to major ill health.	Chief Scientist Office
Yang 2020 (10)	**Intervention:**<br>3-month follow-up: 3 lost to follow-up (because of scheduling problems).<br>**Control:** Usual care.<br>3-month follow-up: 4 lost to follow-up (because of scheduling problems).	No information

NA indicates the study did not have comparators. The comparator group was baseline data.

References

1. Bourke L, Thompson G, Gibson DJ, Daley A, Crank H, Adam I, et al. Pragmatic lifestyle intervention in patients recovering from colon cancer: a randomized controlled pilot study. Archives of physical medicine and rehabilitation. 2011;92(5):749-55.
2. Gordon L, Patrao T, Kularatna S, Hawkes A. A telephone-delivered multiple health behaviour change intervention for colorectal cancer survivors: making the case for cost-effective healthcare. European Journal of Cancer Care. 2015;24(6):854-61.
3. Hawkes AL, Chambers SK, Pakenham KI, Patrao TA, Baade PD, Lynch BM, et al. Effects of a telephone-delivered multiple health behavior change intervention (CanChange) on health and behavioral outcomes in survivors of colorectal cancer: a randomized controlled trial. Journal of Clinical Oncology. 2013;31(18):2313-21.
4. Hawkes AL, Pakenham KI, Chambers SK, Patrao TA, Courneya KS. Effects of a multiple health behavior change intervention for colorectal cancer survivors on psychosocial outcomes and quality of life: a randomized controlled trial. Annals of Behavioral Medicine. 2014;48(3):359-70.
5. Hawkes AL, Patrao TA, Green A, Aitken JF. CanPrevent: a telephone-delivered intervention to reduce multiple behavioural risk factors for colorectal cancer. BMC cancer. 2012;12(1):560.
6. Ho M, Ho JW, Fong DY, Lee C, Macfarlane DJ, Cerin E, et al. Effects of dietary and physical activity interventions on generic and cancer-specific health-related quality of life, anxiety, and depression in colorectal cancer survivors: a randomized controlled trial. Journal of Cancer Survivorship. 2020;14(4):424-33.
7. Lee C, Ho JW, Fong DY, Macfarlane DJ, Cerin E, Lee AM, et al. Dietary and physical activity interventions for colorectal cancer survivors: a randomized controlled trial. Scientific reports. 2018;8(1):5731.
8. Lynch BM, Courneya KS, Sethi P, Patrao TA, Hawkes AL. A randomized controlled trial of a multiple health behavior change intervention delivered to colorectal cancer survivors: effects on sedentary behavior. Cancer. 2014;120(17):2665-72.
9. Macleod M, Steele RJ, O'Carroll RE, Wells M, Campbell A, Sugden JA, et al. Feasibility study to assess the delivery of a lifestyle intervention (TreatWELL) for patients with colorectal cancer undergoing potentially curative treatment. BMJ open. 2018;8(6):e021117.
10. Yang S-Y, Wang J-D, Chang J-H. Occupational therapy to improve quality of life for colorectal cancer survivors: a randomized clinical trial. Supportive Care in Cancer. 2020;28(3):1503-11.
